# Influence of Magnetic Field Strength on Intravoxel Incoherent Motion Parameters in Diffusion MRI of the Calf

**DOI:** 10.3390/tomography10050059

**Published:** 2024-05-17

**Authors:** Tamara Alice Bäuchle, Christoph Martin Stuprich, Martin Loh, Armin Michael Nagel, Michael Uder, Frederik Bernd Laun

**Affiliations:** Institute of Radiology, University Hospital Erlangen, Friedrich-Alexander-Universität Erlangen-Nürnberg (FAU), 91054 Erlangen, Germany; tamara.baeuchle@icloud.com (T.A.B.); christoph.stuprich@uk-erlangen.de (C.M.S.);

**Keywords:** IVIM, diffusion, calf, perfusion, field strength, skeletal muscle

## Abstract

**Background:** The purpose of this study was to investigate the dependence of Intravoxel Incoherent Motion (IVIM) parameters measured in the human calf on B_0_. **Methods:** Diffusion-weighted image data of eight healthy volunteers were acquired using five *b*-values (0–600 s/mm^2^) at rest and after muscle activation at 0.55 and 7 T. The musculus gastrocnemius mediale (GM, activated) was assessed. The perfusion fraction *f* and diffusion coefficient *D* were determined using segmented fits. The dependence on field strength was assessed using Student’s *t*-test for paired samples and the Wilcoxon signed-rank test. A biophysical model built on the three non-exchanging compartments of muscle, venous blood, and arterial blood was used to interpret the data using literature relaxation times. **Results:** The measured perfusion fraction of the GM was significantly lower at 7 T, both for the baseline measurement and after muscle activation. For 0.55 and 7 T, the mean f values were 7.59% and 3.63% at rest, and 14.03% and 6.92% after activation, respectively. The biophysical model estimations for the mean proton-density-weighted perfusion fraction were 3.37% and 6.50% for the non-activated and activated states, respectively. **Conclusions:** B_0_ may have a significant effect on the measured IVIM parameters. The blood relaxation times suggest that 7 T IVIM may be arterial-weighted whereas 0.55 T IVIM may exhibit an approximately equal weighting of arterial and venous blood.

## 1. Introduction

The Intravoxel Incoherent Motion (IVIM) concept described by Le Bihan et al. represents a diffusion-weighted imaging approach that allows for the simultaneous assessment of blood flow and tissue diffusion [1]. The IVIM model assumes that the measured diffusion-weighted signal is represented by one diffusion and one perfusion compartment, which are not in exchange.

For sufficiently low *b*-values, the signal of the diffusion compartment can be described by the following monoexponential signal decay with reasonable approximation [2]:

$$S=S_{0}\exp\left(-bD\right).$$

$S_{0}$ denotes the unweighted signal strength, b is the diffusion weighting (in s/m^2^), and D is the apparent diffusion coefficient of the tissue (in m^2^/s).

Including the perfusion compartment, the signal decay is commonly described by a biexponential function [1,3]:

$$S=S_{0}\cdot \left(f\exp\left(-bD^{*}\right)+\left(1-f\right)\exp\left(-bD\right)\right),$$

where the first summand represents the signal of the perfusion compartment and is characterized by the perfusion fraction f and the pseudo-diffusion coefficient $D^{*}$. The difference in magnitude between D and $D^{*}$ ($D^{*}$ is usually at least one order of magnitude larger than D) enables the differentiation between the blood and tissue compartments.

In skeletal muscle, blood flow depends on the activity state of the muscle and is therefore an attractive target for the acquisition and interpretation of IVIM data [4]. It also allows for assessing perfusion properties, and is sometimes performed in combination with other MRI perfusion mapping techniques. It has been applied to various diseases such as inflammatory myopathies [5], joint disorders [6], dermatomyositis [7], peripheral arterial disease [8], autoimmune myositis and muscular dystrophy [9], and adolescent idiopathic scoliosis [10]. The data interpretation, however, is complicated by the dependence of IVIM parameters on the acquisition setting. For example, a strong dependency of f on the echo time has been reported [11]. Clarifying the dependencies is critical for appropriately correcting the perfusion fraction [12]. Reporting of the IVIM parameters with and without relaxation weighting corrections has been proposed as a best practice by Englund et al. in their recent review on muscle IVIM [4].

Although the importance of the echo time is well-documented, it appears that less attention has been given to potential field-strength dependencies. The differing B_0_ dependencies of the relaxation times among tissue, arterial, and venous blood [13,14,15] may have a considerable impact on the perfusion fraction. Recently, Riexinger et al. evaluated the B_0_ dependency in the liver by comparing IVIM parameters at 1.5 and 3 T, which they measured using identical acquisition parameters, except for the field strength [16]. Notably, they found no significant difference in f. Barbieri et al. performed a similar study and considered the liver, pancreas, spleen, and kidney at 1.5 and 3 T [17]. As they did not keep the echo time fixed, a potential field-strength dependency was difficult to observe. However, if one considers the 1.5 and 3 T data they acquired with similar echo times of 57 and 52 ms using a Sonata and a Trio scanner, they also found no significant B_0_ dependency of f for the liver, pancreas, and spleen, but a dependency was revealed for the kidney.

We are not aware of a similar study investigating the effects of B_0_ on IVIM parameters for the muscle. This study aimed to investigate this dependency. In a meta-analysis, Englund considered 7 studies performed at 1.5 T and 21 performed at 3 T and the “Results of unpaired Student’s *t*-tests used to compare parameters obtained 1.5 T and 3 T found no statistical differences in D (*p* = 0.34), f (*p* = 0.23), or $D^{*}$ (*p* = 0.85)” [4]. Since the influence of the field-strength effects at 1.5 and 3 T appeared to be rather small in the aforementioned studies, we increased the range of considered values and used field strengths of 0.55 and 7 T [18,19,20].

## 2. Materials and Methods

### 2.1. Data Acquisition

MR images were acquired using a 0.55 T MRI scanner (Magnetom FreeMax, Siemens Healthineers, Erlangen, Germany) and a 7 T scanner (Magnetom Terra, Siemens Healthineers, Erlangen, Germany). For the measurements at 0.55 T, a 24-channel receive contour coil and a spine coil were used (both from Siemens Healthineers, Erlangen, Germany). To acquire data at a field strength of 7 T, a 28-channel receive/1-channel transmit knee coil was used (Siemens Healthineers, Erlangen, Germany).

Diffusion-weighted images were obtained using a vendor-provided echo-planar imaging (EPI) diffusion product sequence with diffusion sensitization in the diagonal direction. That is, each physical gradient was switched on with the same gradient strength. Diffusion-weighted images were recorded at five nominal *b*-values: 0 (two averages), 50 (two averages), 100 (two averages), 500 (four averages), and 600 s/mm^2^ (four averages). Fifteen transversal slices with a thickness of 5 mm and 2 mm spacing between slices were placed in the calf. At 0.55 T, the field of view (FoV) was 400 mm × 250 mm and the matrix size was 160 × 100. That is, the FoV was reduced in the phase direction. At 7 T, the FoV was 400 mm × 400 mm and the matrix size was 160 × 160. The additional acquisition parameters were TR = 2800 ms and TE = 56 ms, parallel imaging using the GRAPPA algorithm (acceleration factor of 2, 12 reference lines at 0.55 T, 24 reference lines at 7 T), anterior-posterior phase direction, and phase partial Fourier factor of 6/8; the fat saturation mode was as follows: spectral attenuated inversion recovery, acquisition bandwidth of 1736 Hz/Px, echo spacing of 0.74 ms (at 0.55 T) and 0.64 ms (7 T), and “monopolar” diffusion scheme (i.e., single refocused). The diffusion encoding direction was (1,1,1)^T^ in the scanner coordinate system.

The following vendor-provided data correction algorithms were used: “2D distortion correction” to correct image distortions arising from gradient non-linearities, and at 0.55 T, “dynamic field correction” to correct for image distortions caused by eddy currents.

Anatomical images were obtained using a *T*_1_-weighted turbo spin-echo sequence with the following acquisition parameters: 15 transversal slices, slice thickness of 5 mm, distance factor of 40%, FoV = 350 mm × 215.6 mm (at 0.55 T) and FoV = 200 mm × 160 mm (at 7 T), 0.8 mm × 0.8 mm in-plane voxel size, TR = 1940 ms (at 0.55 T) and TR = 3000 ms (at 7 T), and TE = 14 ms (at 0.55 T) and TE = 9.7 ms (at 7 T).

Eight healthy volunteers (age range: 21–27 years; male/female: 3/5) were recruited. The study was approved by the institutional ethics committee, and written informed consent was obtained from every volunteer prior to the examinations.

**Figure 1:**
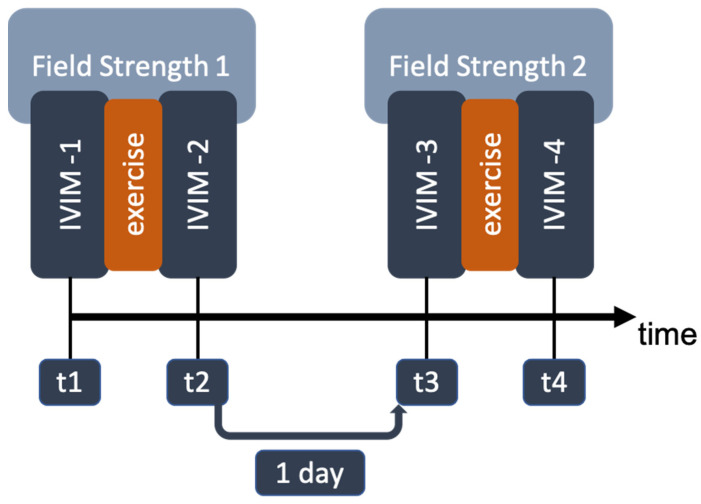
Study design. For each field strength, one baseline measurement (t1, t3) and one measurement after muscle activation (t2, t4) were performed.

To avoid muscle soreness, all volunteers were asked to forgo sports activities for 48 h before the MRI exam. The participants were positioned supine, feet first. The study design was established as follows: first, MRI scans of both lower legs were performed at 0.55 T. The first set of images was acquired. Then, volunteers were instructed to perform 60 s of jumping jacks. Immediately afterward, a second set of images was acquired with identical MR parameter settings. One day later, the 7 T scans were performed (one volunteer was measured first at 7 T). Again, the volunteers were measured twice, once before and once after performing the jumping jacks (Figure 1). Because of the limited space of the knee coil, only one calf of each participant was scanned at 7 T. To keep the time after muscle activation roughly fixed, we decided not to extend the experiment with additional measurements of the other leg. The time between the end of muscle activation and data acquisition was roughly 7 min, and thus, within the time frame where activation was reported to be observable [21]. Sandbags around the legs and the coil were used to minimize volunteer motion during the MRI measurement.

### 2.2. MR Image Analysis

First, a visual check was performed to ensure sufficient data quality and a high enough signal-to-noise ratio (also in the diffusion-weighted images). Manual segmentation of the muscles was performed using the MITK Diffusion (ver. 2017.07.99, German Cancer Research Center (DKFZ), Heidelberg, Germany) application [22]. The following two muscles were assessed: the musculus gastrocnemius mediale (GM) and the musculus tibialis anterior (TA), which is much less affected by the jumping jacks than the GM. For the segmentation of each of the muscles, regions of interest (ROIs) were drawn on all slices of the IVIM *b* = 0 s/mm^2^ images with the help of overlayed anatomical images if necessary. ROIs were carefully placed to exclude fat and large vessels. Then, the ROIs were copied to the images that were acquired with higher *b*-values. The ROI positions were checked and corrected (e.g., if motion had occurred). Next, the mean signal in the ROI was calculated for each acquired image. The calculated mean signals were normalized to the *b* = 0 s/mm^2^ data of the respective image. The equation for the monoexponential IVIM model is expressed as follows:

$$\ln\left(\frac{S\left(b\right)}{S\left(0\right)}\right)=-D\cdot b-f.$$

That is, the diffusion coefficient D was obtained by fitting this equation to the signals at *b*-values ≥ 100 s/mm^2^. The intersection of this monoexponential curve with the signal axis was used to obtain 1 − *f*, where *f* is the perfusion fraction.

The pseudo-diffusion coefficient $D^{*}$ was not computed since this would have made it necessary to acquire *b*-values smaller than 50 s/mm^2^, which was not possible with the used vendor-provided product sequences. Thus, we essentially performed the first step of a segmented fit approach [23,24,25,26,27,28,29]. As an additional evaluation, we performed a Levenberg–Marquardt fit and present the results in the Supplementary Materials.

A signal-to-noise ratio (SNR) analysis was performed using the b800 images (averaged over the four repetitions). One circular ROI approximately 10 cm^2^ in size was placed in the muscle region of the central slice and one circular ROI of approximately equal size was placed in a noise region of the same slice. The SNR was estimated using two methods:

$${SNR}_{1}=\frac{Mean\;signal\;in\;muscle\;region}{mean\;of\;signal\;in\;noise\;region\cdot \sqrt{2/\pi }}$$

$${SNR}_{2}=\frac{Mean\;signal\;in\;muscle\;region}{std\;of\;signal\;in\;noise\;region/\sqrt{2-\pi /2}}$$

The numerical factors $\sqrt{2-\pi /2}$ and $\sqrt{2/\pi }\;$ arise from the single-coil Rayleigh noise distribution approximation [30]. As we used multi-channel coils, the SNR estimates must be regarded as approximations of the true values. Considering that the averaged images were obtained with magnitude averaging, ${SNR}_{1}$ can be regarded as an estimate for the single-repetition images and may teach us about the accuracy and the noise floor. ${SNR}_{2}$ can be regarded as an estimate for the precision in the averaged images. As four averages were used and the SNR generally scales with the square root of averages, one would expect that ${SNR}_{2}\approx 2\cdot {SNR}_{1}$.

### 2.3. Statistics

For each of the fitted IVIM parameters, the Shapiro–Wilk test was used to evaluate normality. According to the result of the Shapiro–Wilk test, either the Wilcoxon signed-rank test or the paired *t*-test was used to detect significant differences between field strengths. The significant level for all statistical tests was set to 0.05.

### 2.4. Plausibility Assessment

We performed the following assessments in order to assess the plausibility of the measured B_0_ dependence of the IVIM parameters. The perfusion fraction f can be calculated as the proportion of the blood signal in the total signal. This relationship is described by the following equation:

$$f=\frac{S_{blood}}{S_{total}}=\frac{f_{0}\cdot w_{b}}{f_{0}w_{b}+(1-f_{0})\cdot w_{m}\;},$$

where $f_{0}$ is the perfusion fraction without relaxation weighting (i.e., proton-density weighted) and the weights of blood and muscle are

$$w_{b}=e^{\frac{-TE}{T_{2,blood}}}\cdot \left(1-e^{\frac{-TR}{T_{1,blood}}}\right)$$

$$w_{m}=e^{\frac{-TE}{T_{2,muscle}}}\cdot \left(1-e^{\frac{-TR}{T_{1,muscle}}}\right).$$

The relaxation times exhibit a dependency on the oxygen saturation level, resulting in different *T*_1_ and *T*_2_ times for oxygenated and deoxygenated blood. The numerical values for venous blood (oxygen level of approximately 72% [31]), arterial blood (oxygen level of approximately 98% [31]), and muscle tissue are shown in Table 1 and Table 2.

The sum of the unweighted perfusion fractions of arterial and venous blood, $f_{0,a}$, and $f_{0,v}$, should equal the total perfusion fraction, $f_{0}$, which is expressed as

$$f_{0}=f_{0,a}+f_{0,v}.$$

Here, as a simplification, the capillary blood pool is not mentioned. It is difficult to state its oxygenation, which varies in this pool. We simply assigned the more oxygenated capillary blood to the arterial blood pool and the less oxygenated capillary blood to the venous blood pool.

The relaxation weights of arterial blood and venous blood, respectively, are

$$w_{a}=e^{\frac{-TE}{T_{2,arterial\;blood}}}\cdot \left(1-e^{\frac{-TR}{T_{1,arterial\;blood}\;}}\right)$$

$$w_{v}=e^{\frac{-TE}{T_{2,venous\;blood}}}\cdot \left(1-e^{\frac{-TR}{T_{1,venous\;blood}}}\right).$$

The relaxation-weighted perfusion fraction becomes

$$f=\frac{S_{blood}}{S_{total}}=\frac{f_{a,0}\cdot w_{a}+f_{v}\cdot w_{v}}{f_{a,0}\cdot w_{a}+f_{v,0}\cdot w_{v}+(1-f_{a,0}-f_{v,0})\cdot w_{m}\;}.$$

**Figure 2:**
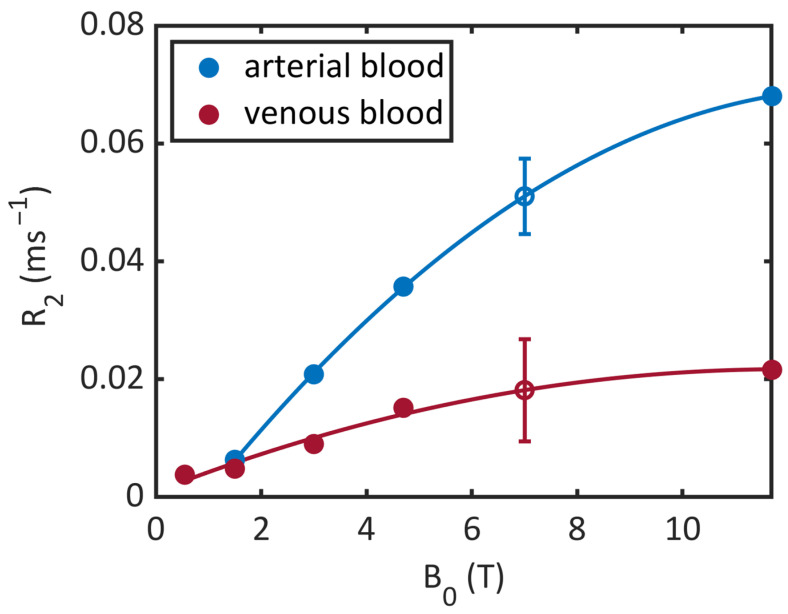
Blood relaxation rates (R_2_). Filled circles denote literature values [14,18,32,33] and the hollow circles denotes the interpolated values for 7 T with a 95 % confidence interval.

To our knowledge, there are no reported values for *T*_2_ times of arterial and venous blood at 0.55 and 7 T. Thus, we estimated the transversal relaxation times for 7 T by considering the relaxation rates reported by Silvennoinen et al. at 1.5 and 4.7 T [14], Zhao et al. at 3 T [32], and Lin et al. at 11.7 T [33], which are shown in Figure 2 for a hematocrit fraction HCT = 0.44. The obtained *T*_2_ times for 7 T shown in Table 1 resulted from an interpolation of the relaxation rates with a second-order polynomial fit function. Unfortunately, the extrapolation to 0.55 T yielded unreasonable results (an almost vanishing relaxation rate for arterial blood). Thus, the physical meaning of the interpolation should not be overvalued; it is essentially just a numerical data-driven approach. According to Campbell-Washburn et al. [18], the *T*_2_ time of arterial blood shows no significant difference between 0.55 T (263 ± 27 ms) and 1.5 T (254–290 ms). Thus, we used literature values of transversal relaxation times of venous and arterial blood at 1.5 T in the context of our study (i.e., 148 ms and 207 ms) [14].

The muscle *T*_2_ time at 7 T that we used in the computations was 22 ms [15]. As we did not find *T*_2_ literature values for 0.55 T for the calf muscle, we assumed that the small difference in *T*_2_ times between the myocardium at 0.55 and 1.5 T reported by Campbell-Washburn et al. translates to the calf muscle [18]. Thus, we used the *T*_2_ time for muscle tissue at 1.5 T reported by Stanisz et al. (i.e., 44 ms) [13].

We used the *T*_1_ time for venous and arterial blood at 7 T reported by Rane et al. (i.e., 2090 and 2990 ms, respectively) [34]. The *T*_1_ time for arterial blood at 0.55 T that we used in the computations was 1122 ms [18]. We did not find *T*_1_ literature values for venous blood at 0.55 T. According to Barth et al., the *T*_1_ time of blood shows virtually no dependency on the oxygenation level at 1.5 T [35]. We assumed that this translates to the longitudinal relaxation time of blood at 0.55 T, and thus, we used the *T*_1_ time for arterial blood at 0.55 T reported by Campbell-Washburn et al. (i.e., 1122 ms) [18].

For the muscle, we used the *T*_1_ time at 7 T reported by Marschar et al. (i.e., 1864 ms) [15]. Unfortunately, we did not find *T*_1_ literature values for the calf muscle at 0.55 T. According to Campbell-Washburn et al. and Stanisz et al., the *T*_1_ time of the myocardium at 1.5 T (950–1030 ms) is similar to the *T*_1_ time of the muscle at 1.5 T (1008 ms) [13,18]. We assumed that the small difference in *T*_1_ times between the myocardium and skeletal muscle translates to those at 0.55 T, and thus, we used the *T*_1_ time of the myocardium at 0.55 T reported by Campbell-Washburn et al. (i.e., 701 ms) [18].

**Table 2:** Longitudinal relaxation times (*T*_1_) for different field strengths and oxygen saturation levels (Y, not stated if not reported in the reference).

	0.55 T	1.5 T	3 T	4.7 T	7 T
Venous blood		1434 ms (Y = 72 %, 23 °C) [35]	1584 ms (Y = 69 %) [36]	1370 ms (Y = 60 %) [37]	2090 ms (Y = 66 %) [34]
Arterial blood	1122 ms [18]	1441–1898 ms [18] 1435 ms (Y = 97 %, 23 °C) [35]	1664 ms (Y = 92 %) [36]	1700 ms (Y = 100 %) [37]	2290 ms (Y = 95.6–99 %) [34]
Muscle		1008 ms [13]	1391 ms [15] 1412 ms [13]		1864 ms [15]
Myocardium	701 ms [18]	950–1030 ms [18]			

## 3. Results

The SNR analysis of the *b* = 800 s/mm^2^ images at 0.55 T yielded an ${SNR}_{1}=7.09\pm 1.89$ and ${SNR}_{2}=11.23\pm 3.16$, where the second number is the standard deviation among volunteers. At 7 T, the analysis could not be performed because the signal in the noise ROI was zero (due to rounding to integers). Given that the signal in the muscle ROIs was around 20, we estimated that the SNR was larger than 20 (at *b* = 800 s/mm^2^).

**Figure 3:**
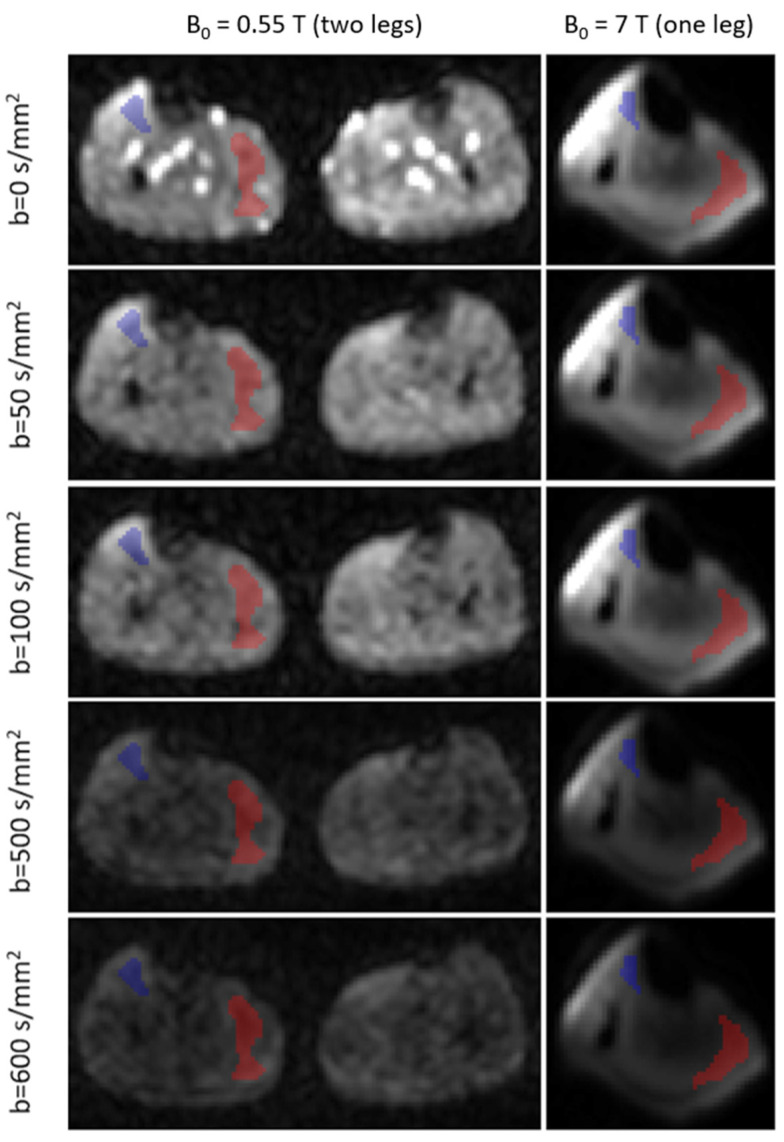
Representative images and ROIs plotted in red and blue colors at different field strengths and *b*-values. Red = GM, musculus gastrocnemius mediale. Blue = TA, musculus tibialis anterior. Note that the images are windowed differently for each field strength. Larger image distortions are visible at 7 T.

Figure 3 shows representative images of one slice of one volunteer acquired at different *b*-values and field strengths (averaged over repetitions). The respective ROIs were plotted, sparing major vessels and fat tissue, and are shown in red and blue colors.

**Figure 4:**
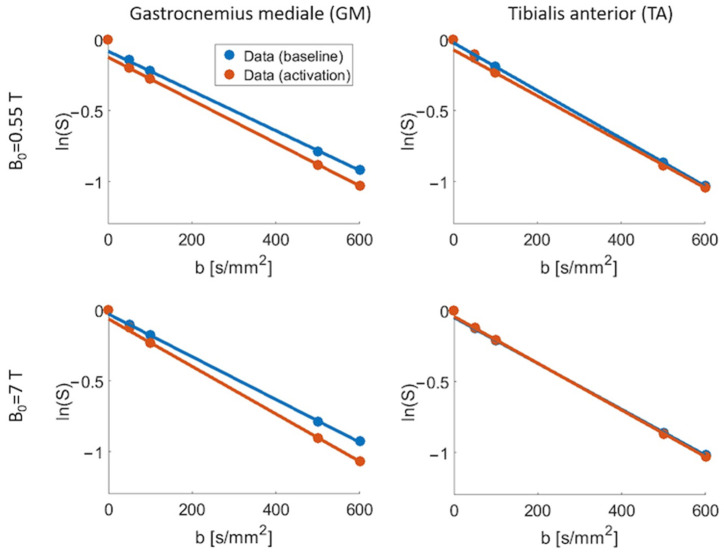
Diffusion-weighted group averaged signal curves obtained for one slice. The monoexponential fit for *b*-values ≥ 100 s/mm^2^ and the intercept with the b=0 axis are depicted for each signal curve. The data points and the respective fit curve that were acquired at rest are plotted in blue. The orange fit curve and data points correspond to the images acquired after the muscle activation.

In Figure 4, representative signal curves are shown, which were measured in different muscle groups at the two field strengths (single-slice data). The monoexponential fit for *b*-values ≥ 100 s/mm^2^ and the intercept with the b=0 axis are depicted for each signal curve. The data points and respective fit that were acquired at rest are plotted in blue. The orange fit and data points correspond to the images acquired after the muscle activation. Both fit curves agree well with the measured data at *b* ≥ 100 s/mm^2^. A decreased perfusion fraction for a higher field strength can be perceived for the GM. There, an increase in the perfusion fraction from baseline to activation can also be observed.

**Figure 5:**
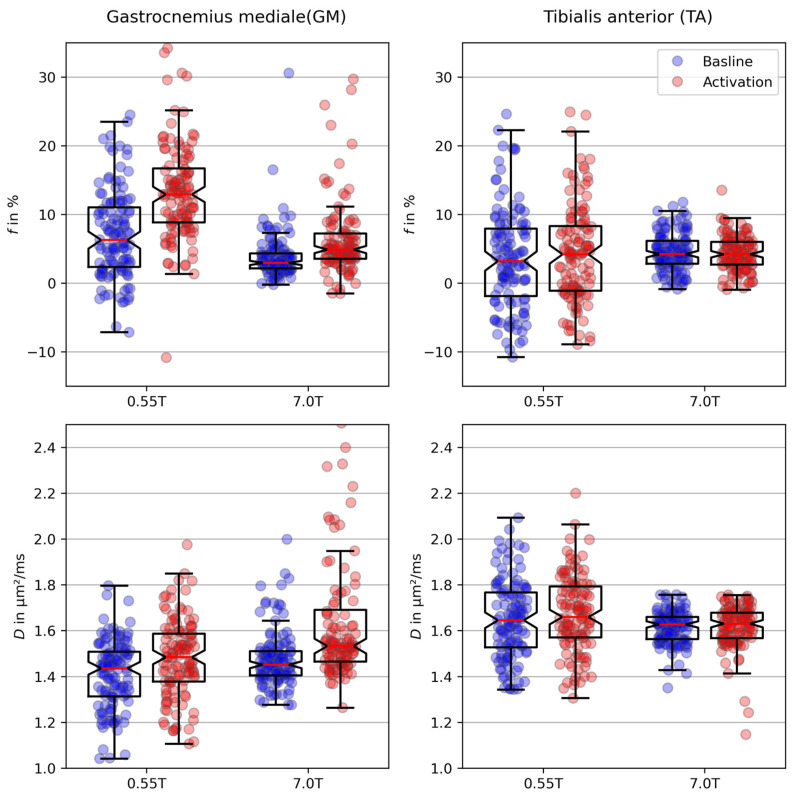
Box plots of the IVIM parameters. Each point represents the parameter obtained for one slice of one volunteer. The central red horizontal line in the box plot indicates the median; the bottom and top lines indicate the 25th and 75th percentiles, respectively. The whiskers reach out to ±1.5*IQR. Not all outliers are shown.

Figure 5 shows the resulting boxplots of the IVIM parameters for both field strengths and muscle groups. Each data point represents the IVIM parameter obtained for one slice of one volunteer. The blue data points indicate the results of the baseline measurement. The orange data points correspond to the results with activated muscles. The mean values and standard deviations are listed in Table 3. The perfusion fraction f of the GM was significantly lower at the higher field strength, both for the baseline measurement (*p <* 0.001) and after muscle activation (*p <* 0.001). For the TA, no significant difference in the perfusion fraction between 0.55 and 7 T was observed (baseline measurement: *p =* 0.14; muscle activation: *p =* 0.59).

The diffusion coefficient D exhibited a significant dependency on B_0_ in the GM after muscle activation, with an increased value at 7 T compared with that at 0.55 T (*p <* 0.001). For the baseline measurement of TA, D was significantly lower at 7 T (*p =* 0.009).

In addition, a significant increase in IVIM parameters from the baseline measurement to activation was observed for the GM at both field strengths (*p <* 0.001). In the TA, a significant increase after muscle activation was only present in the diffusion coefficient at a field strength of 7 T (*p <* 0.003).

**Figure 6:**
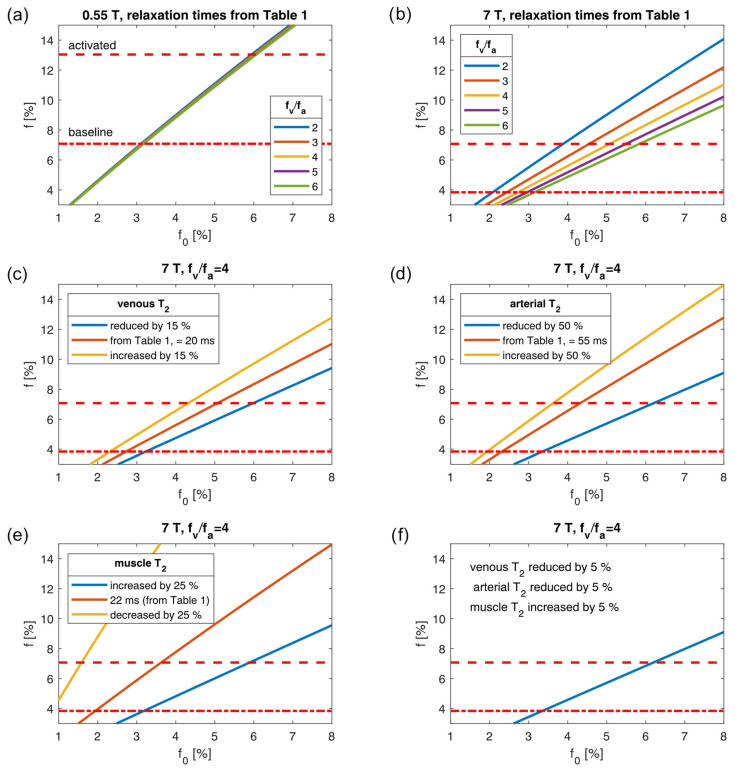
Relaxation-weighted perfusion fraction f as a function of the proton-density-weighted perfusion fraction $f_{0}$ based on the biophysical model. (**a**) At 0.55 T. (**b**) At 7 T. (**c**–**f**) At 7 T with $f_{v}/f_{a}=4$ and variations in the relaxation times. Red horizontal lines: perfusion fractions measured in the GM.

Figure 6a shows the results obtained using the biophysical model and compares them with the perfusion fractions measured in the GM (red lines). The computed relaxation-weighted perfusion fraction *f* is shown for a range of $f_{0}$ values. Since the exact ratio of $f_{v}$ and $f_{a}$ is not known, plots are shown for $f_{v}/f_{a}$ ranging from 2 to 6. The lines for different $f_{v}/f_{a}$ values are similar at 0.55 T because of the small difference between the *T*_2_ of venous and arterial blood. The larger difference between these *T*_2_ times at 7 T leads to a wider separation of the lines (Figure 6b). For this reason, $f_{0}$ can be estimated more precisely at 0.55 T. For the baseline measurement, one can estimate $f_{0}\approx 3.1\;\%$ (using the 0.55 T data, Figure 6a). The same inference can be made for the data obtained after muscle activation, for which, $f_{0}\approx 6.0\;\%$ (again using the 0.55 T plot).

$f_{0}$ should not vary between 0.55 T and 7 T. Figure 6b shows that $f_{0}\approx 3.1\;\%$ (before activation) and 6.0 % (after activation) implies a ratio of arterial and venous blood, $f_{v}/f_{a}$, of approximately six (green line in Figure 6b). This statement holds under the assumption that the used relaxation times, which are stated in Table 1, are appropriate. Since they come with a decent amount of uncertainty, Figure 6c–f explore the variations in relaxation times (for $f_{v}/f_{a}=4$). These plots show that $f_{0}\approx 3.1\%$ (before activation) and 6.0% (after activation) can be reached for $f_{v}/f_{a}=4$ with different settings: 1. with a reduction in the *T*_2_ time of venous blood by 15% (Figure 6c, blue line); 2. with a reduction in *T*_2_ time of the arterial blood by 50% (Figure 6d, blue line); 3. an increase in the muscle *T*_2_ time by 25% (Figure 6e, blue line); 4. a reduction in the blood *T*_2_ times by 5% and a concurrent increase of the muscle *T*_2_ time by 5% (Figure 6f, blue line).

## 4. Discussion

We investigated the influence of the field strength on the IVIM parameters measured in human calf muscles and observed an increase in the perfusion fraction f by a factor of approximately two at 0.55 T compared with 7 T in the activated musculus GM. To our knowledge, this is the first report on the field-strength dependency of measured muscle IVIM parameters. This dependency can be explained by the field-strength dependencies of the relaxation times of muscle tissue and blood. The biophysical model yielded the estimation $f_{0}\approx 3\%$ and 6% in the baseline and activated states, respectively, using the 0.55 T data. It also showed that using 0.55 T and 1.5 T IVIM may be better suited for estimating $f_{0}$ than 3 T and 7 T IVIM because the relaxation times of arterial and venous blood are more similar at a lower B_0_.

Although a large number of muscle IVIM studies have been performed (see Table 1 in the review by Englund et al. [4]), none of them used more than one field strength and fixed parameter settings. Inferring the presence of a B_0_ dependency from the available studies is difficult because of the varying acquisition settings. These include the chosen *b*-values, echo times, and repetition times, as well as the muscle activation procedures and the considered muscles.

Understanding the B_0_ dependencies is necessary to reliably compare studies. Furthermore, using B_0_ to represent a dimension in multi-dimensional diffusion experiments [38,39]—and particularly in IVIM experiments—is appealing for the differing B_0_ dependencies of subcompartments such as the *T*_2_ times of venous and arterial blood. The arterial blood’s *T*_2_ time remains long at 7 T, while the venous blood’s *T*_2_ time shortens drastically (see Table 1). This suggests that 7 T IVIM is “artery-weighted,” whereas low-field IVIM is “blood-weighted (artery and vein-weighted)”; or “vein-weighted”, if the volume fraction of venous blood is much higher than that of arterial blood.

The three-pool model that we used (pools: muscle, arterial, venous) can explain the B_0_ dependency of the measured IVIM parameters and gave reasonable estimates for $f_{0}$. These estimates were based on published (and interpolated) relaxation times and oxygenation levels. The exact values the model predicted should not be overinterpreted as some serious simplifications were made. For example, we did not measure the relaxation times individually for each volunteer, even though, for example, the blood relaxation times are known to depend on the hematocrit (e.g., [14]) and the hematocrit was most likely not identical for all volunteers. Figure 6 shows that slight variations in relaxation times can lead to very different model predictions. This is particularly true at 7 T, where the *T*_2_ times of venous blood and muscle are short compared to the echo time, so that the exponential weighting can change considerably. Nonetheless, the model analysis showed that the observed decrease in f at higher field strengths is indeed plausible and explainable with the model’s predictions. In particular, it is the much longer *T*_2_ time of the arterial blood (in comparison to the muscle *T*_2_ time) that explains the dependency.

If one wanted to add an additional step, one could try to estimate the oxygenation levels as well, but the limited amount of data (f measured at two field strengths → two data points) makes this difficult. Acquiring data with a range of echo and repetition times at varying B_0_ values might help in this regard. Although these estimations were reasonable on a group level, physiological variations in hematocrit and oxygenation levels among various people might make estimations more difficult on an individual level.

It may be natural to assume that similar models could be applied to other organs. However, this is not necessarily the case. For example, the effect of B_0_ dependencies on liver IVIM parameters has been investigated by Riexinger et al., comparing 1.5 and 3 T data [16]. They reported no significant change in f (1.5 T: 28.6%; 3 T: 30.03%). There appears to be a contradiction between the liver experiments performed by Riexinger et al. (no B_0_ dependency, similar to Barbieri et al. [17]) and the muscle experiments in the current study (strong B_0_ dependency). If one applies the two-pool model to the liver with TE = 100 ms, as conducted by Riexinger et al., the liver relaxation times reported by De Bazelaire et al. (*T*_2_: 34 ms at 3 T, 46 ms at 1.5 T; *T*_1_: 586 ms at 1.5 T, 809 ms at 3 T) [40], and the blood relaxation times of Table 1 and Table 2, then the presence of a noticeable B_0_ dependency may be expected, but only if the ratio of venous to arterial blood volume equals four ($f_{1.5T}/f_{3T}\approx 1.27$, see Supporting Information). This discrepancy disappears if one assumes equal volumes of arterial and venous blood ($f_{3T}/f_{1.5T}\approx 1.00$). Since we would rather assume a volume ratio of four than one, it is not clear why a *T*_1_ dependency was not observed for the liver but was observed for the muscle. Potentially, some effects could make IVIM liver experiments more sensitive to arterial blood. Further research is necessary in this regard, particularly with larger spans of B_0_ values within a single study while keeping all other acquisition parameters fixed.

It should be noted, however, that acquiring data with a wide range of B_0_ values involves several challenges. Low-field MR data generally suffer from a reduced signal-to-noise ratio [18,19]. We also observed a reduced SNR at 0.55 T, but it was high enough that a noise floor correction was not necessary. The ratio between the two SNR estimates, ${SNR}_{2}/{SNR}_{1}$, was approximately 1.6 and not 2 as one would expect for a single-coil setup. We attribute this difference to the multi-channel coil setup. Since the IVIM fit eventually becomes non-linear, noise can cause a bias in the IVIM parameters that arises from the scatter of the data (cf., e.g., [41]). As we used the ROI-averaged signal in our analysis, the SNR of the ROI-averaged signal must be considered (with respect to this effect), which scales as ${SNR}_{ROI}={SNR}_{singlevoxel}\cdot \sqrt{N_{ROI}}$, where $N_{ROI}$ is the number of voxels in the ROI. For example, for the GM and our 0.55 T data, the mean $N_{ROI}$ was 23 and hence $\sqrt{N_{ROI}}\approx 5$. Thus, the SNR at *b* = 800/smm^2^ for this setting was effectively $\approx {SNR}_{1}\cdot \sqrt{N_{ROI}}\approx 7\cdot 5=35$, and therefore, presumably not within a problematic range. Another effect is that fat saturation efficiency might vary between field strengths. However, we did not note such an effect and care was taken to avoid any visible fat signal (which usually is easily visible as a ring-like structure of the subcutaneous fat) so we assumed that varying fat saturation levels have a minor effect on our evaluation. Ultra-high field MRI exams are prone to inhomogeneities in the transmitted magnetic field (B_1_^+^) and main magnetic field (B_0_). The latter results in increased spatial distortions in echo-planar images [20]. The resulting flip angle inhomogeneity can be mitigated by parallel transmission techniques [42]. The use of readout-segmented EPI or interleaved EPI, for example, can considerably reduce image distortions [43,44]. Nonetheless, we used a potentially more robust and faster conventional EPI sequence because the image distortions did not hamper our ROI-based evaluation.

The increase in D that we observed after activation has been reported previously [45,46,47], which may be due to an increase in temperature. An increase in D by 5%, which is roughly what we observed, would correspond to a temperature change of approximately 2 °C for free water [48,49]. This is a reasonable temperature change in the extremities after activation. Nonetheless, further research might be warranted in this regard, using a range of diffusion times to reveal potential changes in membrane permeability [50].

The B_0_ dependency of D was ambiguous because D increased for the GM but decreased for the TA. This inconsistency may indicate a small effect size and implies that it is unlikely that the muscle tissue compartment was more accurately described by additional pools [38,51].

The used biophysical model might also be used in experiments involving multiple TE and TR times to retrieve the model parameters, for example. The main focus of our current study, however, was less focused on achieving a fit of these model parameters, and more focused on investigating whether a field strength dependency exists for IVIM exams of the calf muscles. It is appealing, however, to perform IVIM experiments with multiple TEs and B_0_ values in future studies.

There were also several limitations to our study. We could not acquire data with small *b*-values (<50 s/mm^2^). We did not have access to a sequence coding environment for the newly installed 0.55 T scanner, so we had to rely on the vendor-provided diffusion-weighted EPI sequence. This sequence provided a minimum step size of 50 s/mm^2^ for the *b*-values. For that reason, we did not obtain estimates of $D^{*}$, as they would not have been reliable. Moreover, the tri-exponential behavior reported for other organs could not be assessed for this reason [16,29,52,53,54]. A further limitation is that we only used 5 *b*-values and the use of more *b*-values might have increased the precision and accuracy. Fitting IVIM parameters is notoriously difficult. For example, Wurnig et al. proposed higher threshold cutoffs than *b* = 100 s/mm^2^, which was used in our study [55,56]. Using, for example, a different cutoff value may also affect our parameter estimates. Another potential limitation was our small sample size of eight. Although a larger number might have increased the statistical certainty, we were well in the range commonly used in methodological MRI studies [57]. Another limitation was the use of only two field strengths, 0.55 and 7 T. Acquiring data at 1.5 and 3 T would likely have been valuable, but we omitted these scans to limit the burden for the volunteers. One limitation of the biophysical model is the wide range of reported *T*_1_ and *T*_2_ values. For example, for *T*_2_ of the muscle, Stanisz et al. reported *T*_2_ ≈ 44 ms at 1.5 T and Gold et al. reported 35.3 ms [58]. Moreover, the blood relaxation times depend heavily on the hematocrit [32], which varies among individuals [59]. Thus, the estimates of $f_{0}$ strongly depends on choosing the correct relaxation times. A potential solution is the additional measurement of individual relaxation times. A further limitation may be that we did not acquire the *b*-value of 200 s/mm^2^, as previous studies used *b*-values ≥ 200 s/mm^2^ (or >200 s/mm^2^) for the segmented fit [60,61,62], whereas we used b≥ 100 s/mm^2^ for the segmented fit. Also, we did not take into account internal field gradients, which may affect the diffusion measurement [63]. Lastly, the jumping jacks exercise we used for muscle activation is potentially less standardizable than previously used exercises such as, for example, heel raises [45], due to varying jumping intensity among volunteers. Nonetheless, we used the jumping jack exercise because it was easy to perform and preparatory experiments showed that it inflicted little soreness, edema, or other MRI-visible muscle changes, which we wanted to avoid in the ensuing 7 T exams.

## 5. Conclusions

In conclusion, we found a strong dependency of the perfusion fraction f on the magnetic field strength B_0_ in human calf muscles, which can be explained with a biophysical model that accounts for relaxation times. This dependency is relevant when comparing quantitative IVIM parameters among studies and indicates that relaxation-compensated values should be reported, as suggested previously [4]. In this regard, the difference between venous and arterial blood relaxation times at higher field strengths presumably makes it necessary to consider these two pools separately when computing the relaxation compensation, and may lead to an “arterial” weighting. IVIM imaging at 0.55 T, however, may be interpreted as “blood”-weighted with approximately equal weighting of arterial and venous blood.

## Figures and Tables

**Table 1:** Transversal relaxation times (*T*_2_) for different field strengths and oxygen saturation levels (Y, not stated if not reported in the reference).

	0.55 T	1.5 T	3 T	4.7 T	7 T
Venous blood		148 ms (Y = 72 %) [14]	48 ms (Y = 72 %) [32]	28 ms (Y = 72 %) [14]	20 ms
Arterial blood	263 ms [12]	207 ms (Y = 98 %) [14] 254–290 ms [18]	111 ms (Y = 98 %) [32]	66 ms (Y = 98 %) [14]	55 ms
Muscle		44 ms [13] 35.3 ms [33]	31.7 ms [33] 27 ms [9]		22 ms [15]
Myocardium	58 ms [18]	40–58 ms [18]			

**Table 3:** Mean value of the IVIM parameters. GM = musculus gastrocnemius mediale. TA = musculus tibialis anterior.

	B_0_ (T)	GM_Baseline_	GM_Activation_	TA_Baseline_	TA_Activation_
f (%)	0.55	7.08 (±6.41)	13.40 (±7.34)	3.62 (±7.33)	4.06 (±7.45)
	7	3.84 (±3.50)	7.07 (±8.08)	4.53 (±2.73)	4.28 (±2.50)
D (µm^2^/ms)	0.55	1.41 (±0.15)	1.47 (±0.18)	1.65 (±0.17)	1.67 (±0.17)
	7	1.47 (±0.13)	1.67 (±0.37)	1.61 (±0.07)	1.61 (±0.09)

## Data Availability

The datasets generated and analyzed during the current study are not publicly available due to the general data protection regulation (GDPR) but are available from the corresponding author upon reasonable request.

## Supplementary Materials

The following supporting information can be downloaded at: https://www.mdpi.com/article/10.3390/tomography10050059/s1. Levenberg–Marquardt fit results: “Levenberg–Marquardt fits.pdf”. Matlab code M1: “Supplemental_Information_Muscle.m”: the biophysical model for the muscle. Matlab code M2: “Supplemental_Information_Liver.m”: the biophysical model for the liver.
