# Supplementary material for: Influence of Magnetic Field Strength on Intravoxel Incoherent Motion Parameters in Diffusion MRI of the Calf
Source: Tomography. 2024 May 17;10(5):773–88. doi: 10.3390/tomography10050059 (PMC11126135; doi:10.3390/tomography10050059)

Here, we present the results obtained with the Levenberg-Marquardt fit. The fit was initialized with $S_0 = 40$, $f = 0.01$, $D = 1$ µm²/ms, and $D^* = 10$ µm²/ms. Since the standard deviation of the fitted parameters was much larger than for the segmented fit, the Table shows the median values. The fit nonetheless supports the main finding f is considerably smaller at 7 T.

	**B_0 (T)**	$GM_{Baseline}$	$GM_{Activation}$	$TA_{Baseline}$	$TA_{Activation}$
f (%)	0.55	8.37	14.31	4.51	5.84
	7	3.13	4.85	4.41	4.55
D (µm²/ms)	0.55	1.35	1.46	1.64	1.62
	7	1.45	1.52	1.63	1.62

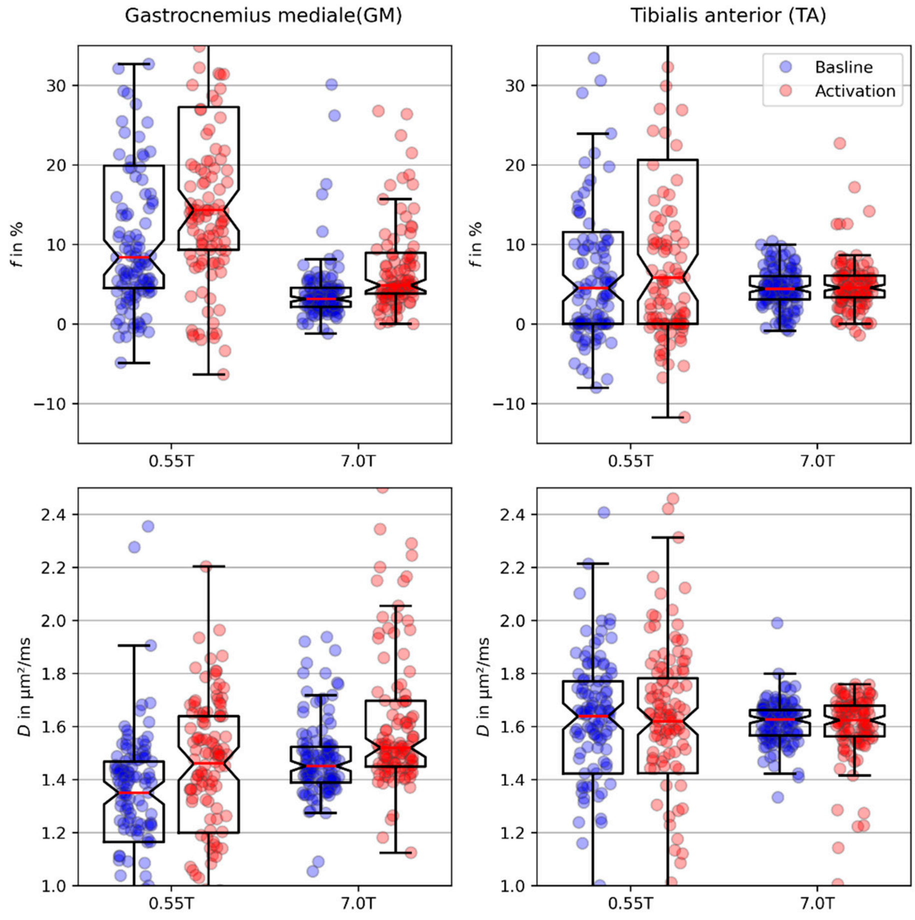

Supplement: Supplementary file 1 [file tomography-10-00059-s001.zip › Levenberg¿CMarquardt fits.pdf]
